# Penetrance estimates of hereditary cancers in a population setting using UK Biobank data

**DOI:** 10.1038/s44276-023-00021-x

**Published:** 2024-03-15

**Authors:** Ranjit Manchanda, D. Gareth Evans, Antonis C. Antoniou, Adam Brentnall

**Affiliations:** 1 Wolfson Institute of Population Health, Queen Mary University of London, London, EC1M 6BQ UK; 2 Department of Health Services Research and Policy, London School of Hygiene & Tropical Medicine, London, WC1H 9SH UK; 3 Department of Gynaecological Oncology, Barts Health NHS Trust, Royal London Hospital, London, E1 1BB UK; 4 Manchester Centre for Genomic Medicine, Division of Evolution, infection and Genomic Sciences, University of Manchester, MAHSC, 6th Floor Saint Mary’s Hospital, Manchester, UK; 5 Centre for Cancer Genetic Epidemiology, Department of Public Health and Primary Care, University of Cambridge, Cambridge, UK

**arising from** L. Jackson et al. eClinicalMedicine 10.1016/j.eclinm.2023.102159 (2023)

Average cancer risks for *BRCA1/BRCA2* and Lynch Syndrome pathogenic-variant carriers are above established thresholds of clinical intervention for patient benefit, even in those without a cancer family-history. A recent analysis based on UK-Biobank data suggesting that risk in carriers is primarily conferred by cancer family-history has multiple interpretation and methodological flaws.

*BRCA1, BRCA2*, and mismatch repair (Lynch Syndrome) cancer susceptibility genes (CSGs) fall under Tier-1 genomic applications (as defined by the Centres for Disease Control, Office of Public Health Genomics) which have significant potential for positive impact on public health based on available evidence-based guidelines and recommendations [1–3]. Testing for these CSGs has been evaluated to have clinical utility by NICE and other professional bodies, where subsequent interventions including for early detection (screening) and prevention or risk reduction recommended in published guidelines, could significantly reduce morbidity and mortality [3–6]. Previous consortia-based studies analysed data from diverse settings to estimate cancer risks for pathogenic variant (PV) carriers of these CSGs. These demonstrated modification of cancer risks by family history (FH), but the average risks for those without cancer FH were still above established intervention thresholds [7–9].

A recent report from Jackson et al. [10] estimated breast and colorectal cancer risks for PV carriers in *BRCA1/BRCA2* and Lynch-Syndrome genes, using data from the UK-Biobank cohort. This cohort is relatively old at entry, with recruitment ages of 40-to-69 years, and healthy. Their main finding that the cancer risks are modified by cancer family-history (FH), is in agreement with the previous reports [8]. However, their analysis contains multiple statistical methodological issues, and their interpretation of their estimates raises several concerns. Additionally, some of the comparisons in the article and communications are incorrect [11]. Anecdotally, this has led to concern amongst stakeholders including healthcare professionals who counsel on cancer risk management and patients.

From a methodological perspective their Kaplan-Meier and Cox regression analyses considered retrospective and prospective data from UK-Biobank, treating the cohort as if they were enroled at birth. This is not true and this approach leads to high risk of bias due to beginning follow-up prior to when participants joined the cohort. For example, individuals who had cancer before entry, or were undergoing current cancer treatment, are under-represented in comparison to the general population. In fact, individuals who had cancer and died before the study’s commencement may not be included in the analysis. These factors make the study at risk of producing lower than expected estimates of risk, particularly at ages younger than 40, which cannot reliably estimated. A further limitation is the assumption of a constant relative-hazard for PV carriers across all age-groups. Given the older age of the UK-Biobank cohort (mean age = 56 years), the relative hazard estimates are likely heavily dominated by effects in older age-groups, overlooking well-established findings that *BRCA1*/*BRCA*2 BC relative-risks decrease with age [8].

UK-Biobank is a highly selected cohort with a strong healthy volunteer effect. For example, at age 70–74 y rates of all-cause mortality were 46–56% lower and total cancer incidence 12–18% lower than the general population [12]. Therefore, estimates may further be susceptible to selection biases.

When drawing wider conclusions about the results it is important to make right comparisons from an analysis. “The authors’ interpretation of the 20-year risk between 40–60 as cumulative-risks to a specific age and their comparisons to “lifetime-risks” in figure-2 and the discussion in their manuscript [10], along-with the press release, are misleading. They overlook the well-established high BC incidence in *BRCA1/BRCA2-*carriers under 40-years [8, 9], and use inconsistent age-intervals for their comparisons. Clearly by definition their estimates will be lower than lifetime risk. It is incorrect to compare risk-estimates for the 40–60 year interval to established intervention thresholds based on lifetime-risks and to intimate that *BRCA1/BRCA2* PV-carriers do not meet NICE guideline high-risk threshold without FH.

Their conclusion that “*much of the risk conferred by a rare PV associated with HBOC ……. is conferred by a FDR family-history of disease*” is not supported by their results. For example, their estimated relative-hazard estimate of 7.2 among *BRCA1-*carriers without FH translates to an approximate 60% BC-risk by age 80 y, assuming the latest UK-population BC incidences [13]. This is well above the NICE and other well-established thresholds for clinical intervention [3] and similar to previous risk estimates by FH [8].

Their unaddressed methodological issues are compounded by the interpretation of their results based on a small number of PV carriers. Ignoring larger datasets (with many more PV carriers) that have considered the issue regarding FH and provide reliable analysis in their interpretation is imprudent [8].

In conclusion, comprehensive risk assessment in carriers should include FH. This should be done using established risk prediction models [14], in conjunction with recognised NICE and guideline based clinical intervention thresholds [3, 5, 6].
